# Migratory management and environmental conditions affect lifespan and oxidative stress in honey bees

**DOI:** 10.1038/srep32023

**Published:** 2016-08-24

**Authors:** Michael Simone-Finstrom, Hongmei Li-Byarlay, Ming H. Huang, Micheline K. Strand, Olav Rueppell, David R. Tarpy

**Affiliations:** 1 Department of Entomology, North Carolina State University, Raleigh, NC, USA; 2 The W.M. Keck Center for Behavioral Biology, North Carolina State University, Raleigh, NC, USA; 3 Department of Biology, University of North Carolina at Greensboro, Greensboro, NC, USA; 4 Life Sciences Division, U.S. Army Research Office, Research Triangle Park, NC, USA.

## Abstract

Most pollination in large-scale agriculture is dependent on managed colonies of a single species, the honey bee *Apis mellifera*. More than 1 million hives are transported to California each year just to pollinate the almonds, and bees are trucked across the country for various cropping systems. Concerns have been raised about whether such “migratory management” causes bees undue stress; however to date there have been no longer-term studies rigorously addressing whether migratory management is detrimental to bee health. To address this issue, we conducted field experiments comparing bees from commercial and experimental migratory beekeeping operations to those from stationary colonies to quantify effects on lifespan, colony health and productivity, and levels of oxidative damage for individual bees. We detected a significant decrease in lifespan of migratory adult bees relative to stationary bees. We also found that migration affected oxidative stress levels in honey bees, but that food scarcity had an even larger impact; some detrimental effects of migration may be alleviated by a greater abundance of forage. In addition, rearing conditions affect levels of oxidative damage incurred as adults. This is the first comprehensive study on impacts of migratory management on the health and oxidative stress of honey bees.

Honey bees (*Apis mellifera*) are the most economically important pollinators in North America and are crucial for sustaining production in many agroecosystems^1^. Honey bee colonies are composed of tens of thousands of individuals, which allows them to pollinate crops effectively over a large geographic area, particularly with the assistance of beekeepers who transport colonies for pollination services. The major economic driver of the beekeeping industry in the U.S. is fulfilling pollination contracts for various growers, including almonds, berries, apples, and cucurbits^2^. Therefore, commercial beekeepers transport bee colonies on trucks both regionally and nationally for many months of the year^3^,^4^,^5^. Given this paradigm, many colonies are repeatedly moved over several months to a series of large monocultures, which potentially increases a colony’s exposure to pesticides^3^,^6^ and pathogens^7^, limits access to diversified pollen sources^8^, and forces the foraging bees to re-learn and re-assess their environmental surroundings. The assumption, therefore, is that factors associated with migratory beekeeping operations overwhelm bees and induce a stress response, ultimately contributing to increased colony losses and susceptibility to disease, parasites, and syndrome-like effects such as Colony Collapse Disorder (CCD)^9^.

However, the impact of transporting colonies for pollination services has currently received minimal investigation^7^,^10^. Furthermore, no study has empirically examined the cellular consequences of stress to honey bee workers under this paradigm. On a physiological level, the immediate effect of transportation (i.e., 24-hours after a 3-day trip) has been shown to lead to a reduction in the size of the glands that are essential for brood food production in nurse bees^10^. However, the long-term implications of this finding are unclear. Thus, the goal of this study was to better resolve the question regarding whether migratory beekeeping practices cause changes in measurable levels of oxidative stress and possible impacts of the cells throughout a season.

Our ability to understand and quantify stress is critical for evaluating the impacts of abiotic and biotic factors influencing honey bee health and colony productivity. Oxidative stress is important in eukaryotic organisms and can have severe negative effects. Reactive oxygen species (ROS) are the causative agents of oxidative stress, and they are produced as a by-product of normal metabolic processes (or otherwise suffer from diminished redox homeostasis). Cells that lose their ability to remove excess ROS undergo oxidative stress, which leads to DNA mutation^11^, irreparable damage of proteins^12^, and membrane instability^13^. Oxidative stress can lead to apoptosis and cellular damage, which are intimately linked to aging^14^,^15^. Acute exposure to mild stress can extend lifespan because stress-resistance mechanisms, like the production of antioxidants, can be activated^16^. However, severe or chronic stressors, like prolonged sublethal pesticide exposure^17^, usually shorten lifespan^18^. In particular, some theories argue that aging is simply a result of the accumulation of oxidative damage^19^,^20^. Furthermore, ROS may be induced by exogenous sources (i.e. pesticides and environment). We hypothesized that migratory honey bees experience oxidative stress and may have shorter life spans.

The biomarker malondialdehyde (MDA) is a common measure of oxidative stress in honey bees, other insects, and vertebrate systems^21^,^22^,^23^,^24^. MDA is the main organic compound produced from lipid peroxidation of polyunsaturated fatty acids in cellular membranes^25^. MDA levels reflect the combined effects of exposure to oxidative stress and the ability or lack thereof to resist oxidative damage through various repair mechanisms^26^.

We determined how the movement of managed honey bee colonies across different agricultural landscapes influenced colony health and productivity, adult lifespan, and levels of oxidative stress, measured as MDA. Our study is the first to examine the long-term effects of migratory colony management on stress accumulation in honey bees. This study was conducted in three parts where we: 1) determined the effect of migratory management on honey bee lifespan; 2) investigated the effects of migratory management on colony health, productivity, lifespan, and oxidative stress on either stationary or migratory bees; and 3) investigated the effects of intensive, short-term migratory movement on levels of oxidative stress in honey bees.

## Methods

**Figure 1:**
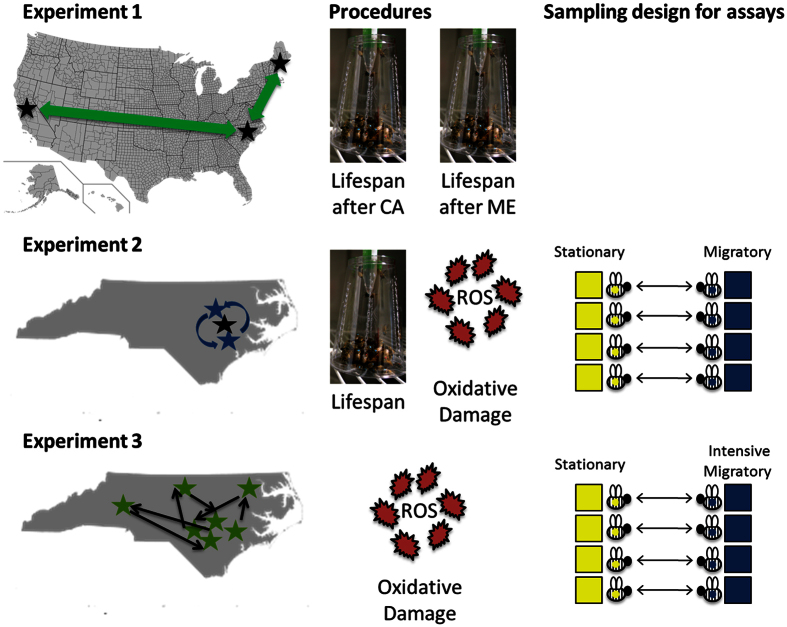
The experimental schemes of Experiment 1, 2, and 3. The first panel depicts where the bees were transported for the different experiments, the second shows the procedures used (lifespan analysis in incubator cages or oxidative stress analyses), and the third depicts the paired design aspect where colonies were matched, and newly emerged bees were paint-marked and swapped across these pairs. This figure was created in Microsoft Powerpoint. Pictures were taken by and all items designed or modified by M. Simone-Finstrom. Maps were modified from figures available through Wikimedia Commons. The original US map is available at https://en.wikipedia.org/wiki/File:USA_Counties.svg, and the original NC map can be found at https://commons.wikimedia.org/wiki/File:Bluenc.png; both are in the public domain.

A scheme of the general experimental designs for Experiments 1, 2, and 3 is shown in Fig. 1.

### Experiment 1: Effects of commercial migratory operation on bee lifespan

To remove the confounding effects of the hive environment and energetic costs related to foraging behavior, the lifespan of worker bees from stationary and migratory colonies was determined under controlled conditions following standard protocols^27^. Frames of emerging workers were collected from 8 colonies from a large apiary in Henderson, NC shortly after completing pollination services in California (~4,500 km distance). An additional 8 colonies were chosen of comparable population maintained at the Lake Wheeler Farms Bee Research Facility at North Carolina State University, Raleigh, NC to represent stationary hives. Following established methods^27^, ~45 newly emerged workers from each of the 16 colonies were color-marked using Testors enamel paint and divided evenly among 12 plastic cups, so that a total of ~60 bees were maintained in each cup. Mortality of color-marked bees was recorded daily. Since small cage populations tend to decline more rapidly, populations were replenished with newly emerged worker bees from a non-experimental source to maintain ≥30 bees per cage at all times. Bees were fed 50% sucrose solution *ad libitum*.

Lifespan analyses were conducted twice during the experimental period (Fig. 1). After the bees had been collected for the first trial in May 2012 (early season), the colonies were transported to Maine for subsequent pollination of lowbush blueberry. Colonies returned to North Carolina and frames containing emerging bees were collected again for a subsequent trial in late June 2012 (late season). Two colonies could not be used for the second trial, as one became queenless and one died, so two other colonies were used. Differences in adult worker lifespan were compared using the parametric survival analysis JMP Pro 10 with colony treatment (i.e., stationary versus migratory) and season (early versus late) as factors in a general linear model.

### Experiment 2: Effects of experimental migratory management on colony demography, bee lifespan, and oxidative stress

#### Colony treatments

Colonies were established from queenless divisions of overwintered hives and initiated in a single bee yard at the Lake Wheeler Farms Bee Research Facility at North Carolina State University, Raleigh, NC in April 2012. Each split was made to contain three brood frames (two sealed, one open), one honey frame, one pollen and five foundation frames (wire with wax). Sister queens were reared from a single Italian queen, placed as virgins within these colonies and allowed to mate naturally. In this way, all colonies had similar genetic backgrounds.

Each colony was maintained in a single Langstroth hive body containing nine frames of comb for the duration of the study. Each colony was given 1–2 extra boxes (“honey supers”) above a queen excluder when needed to ensure it maintained only one box with brood but had enough space to grow to deter swarming. Otherwise, standard beekeeping practices were followed as in the first experiment.

On May 1, 2012 (early season) all colonies were matched for size based on the numbers of frames of bees and brood before the start of the experiment and divided into two treatments. Stationary colonies (N = 9) were maintained in a single apiary bordering a forested park (Yates Mill) and agricultural fields containing mainly corn and wheat. Migratory colonies (N = 10) were initially moved to the Central Crops Research Station in Clayton, NC. Every 21 days (equivalent to one honey bee brood developmental cycle), the migratory colonies were moved among the North Carolina Department of Agriculture Research Station in Goldsboro, NC or Rocky Mount, NC, so that the colonies were moved 35–60 miles on each of five trips (see Supplemental Methods, Tables S1 and S2, for more information on the locations and primary crops). This continued from May 4, 2012 until the final move back to Clayton, NC on July 27, 2012, where the hives remained until the conclusion of the study.

To assess colony growth and general health, queen status, amount of stored pollen, some adult worker bees, and amount of capped brood were measured in May, June, July, and August 2012 following standard protocols^28^. Infestation of the ectoparasite *Varroa destructor* on adult bees was determined at the end of the study period by sampling 300 bees per colony into 95% ethanol to dislodge the mites from the bees, following standard methods^29^. Five colonies (two stationary and three migratory) died during the experiment and were excluded from all colony status analyses, resulting in 7 colonies per treatment. A repeated-measures ANOVA was used to examine differences in colony status between stationary and migratory colonies during the study period.

#### Lifespan analysis

To remove the confounding effects of the hive environment and energetic costs related to foraging behavior, the lifespan of workers from the stationary or migratory colonies was determined under controlled conditions as described for Experiment 1. Lifespan analysis was conducted once in July towards the end of the experimental period and after the colonies had been transported 4 times. Differences in lifespan between workers from stationary and migratory colonies were analyzed as described for Experiment 1.

#### Analysis of oxidative stress (lipid peroxidation via MDA)

Newly emerged worker bees were collected from each colony and paint-marked in May 2012 (before the 2^nd^ move, early season) and in July 2012 (before the 5^th^ move, late season). A paired-colony cross-fostering design, where a portion of these newly emerged, painted adult bees was swapped between a paired colony from the opposite treatment, enabled a separate assessment of the effects of rearing (larval) environment and adult environment (Fig. 1). Paint-marked bees were sampled at 14 and 28 days old (‘age’) either from a brood frame as hive bees or outside the colony entrance as active foragers (‘collection type’). Bees were stored at −80 °C until analyses for oxidative stress.

ROS-mediated oxidative damage was quantified by measuring MDA level in individual worker heads. The assay was conducted using the OXItek™ Thiobarbituric Acid Reactive Substances (TBARS) Assay Kit (ZeptoMetrix Corp). The head of each bee was flash-frozen in liquid nitrogen and immediately pulverized in a 1.5 mL micro-centrifuge tube with a sterile plastic pestle. The tissue was then mixed with 280 μL of PBS, vortexed to homogenize the cellular suspension, and centrifuged briefly to precipitate pieces of tissue and cuticle. Aliquots of the supernatant were used subsequently in the quantification assays. Both the TBARS and the Pierce^™^ BCA^™^ Protein Assay kits (Thermo Scientific) were used according to the manufacturers’ recommendations. Total soluble protein was determined by BCA Protein Assay and used to normalize the corresponding TBARS amounts. Oxidative damage as measured by normalized MDA levels was examined in three biological replicates (colony) in either early season (May) or late season (July). Each condition (rearing and adult environment) included ≥6 individual bees, and a total of 282 bees were tested. A greater number of hive bees (N = 210) were assayed than foragers (N = 72) because of the low availability of foragers at the end of the experiments.

Data were analyzed using a three-way ANOVA to examine if differences in oxidative stress (level of MDA) were due to effects of rearing environment, adult environment, and season (May versus July). Colony effect was considered as a random factor. Tukey-Kramer post-hoc tests were used to make pair-wise comparisons of different experimental groups. Differences were considered significant at α = 0.05.

### Experiment 3: Effect of intensive migratory management on oxidative stress

#### Colony treatment

An independent set of 3 colony pairs (6 hives total) was selected from the Lake Wheeler Farms Bee Research Facility at North Carolina State University, Raleigh, NC in August, 2012. Each colony was maintained in a single brood box (as described for Experiment 2) and trucked 3 hours daily for 6 consecutive days to novel locations within North Carolina. The colonies were moved each night, then opened upon arrival so that they could forage in the new location during the day before they were moved again. The trips were, on average, 218 miles with a range of 205–232 miles (see Supplemental Methods, Table S3, for more information on the locations).

Seven days prior to the first move, newly emerged bees were paint-marked and returned to their hive in addition to a paired stationary hive from Experiment 2. The day after the final move, 14d-old bees were collected from each hive and stored at −80 °C for subsequent measures of oxidative stress as described above.

Due to logistics of driving these colonies around daily, we were only able to fit six colonies on our truck. So we included the maximum number of colonies that we possibly could given the available equipment. For each colony, we have sufficient samples (individuals per colony) for our analyses.

#### Measure of oxidative stress (lipid peroxidation via MDA)

The procedure and statistical analyses were the same as described for Experiment 2. Three colony pairs were sampled, and at least six bees from each treatment group were analyzed (rearing environment, adult environment, age, and collection type), resulting in a total of 185 bees.

## Results

### Experiment 1: Effects of commercial migratory operation

**Figure 2:**
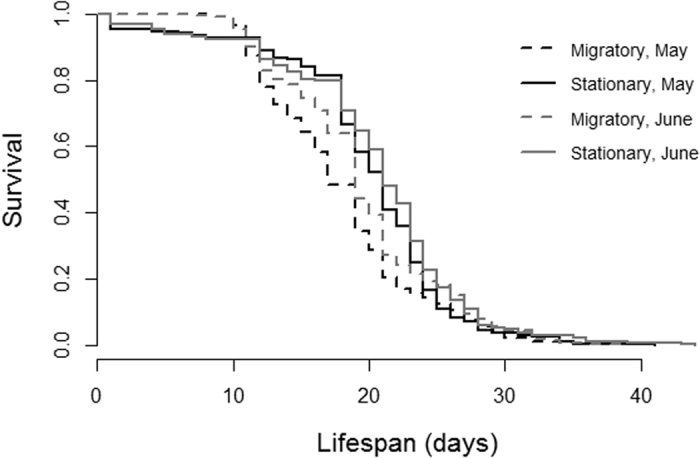
In Experiment 1, bees reared in a commercial, migratory beekeeping operation (dashed line) had reduced lifespan compared to bees reared in a stationary operation (solid lines; *X*^2^ = 11.39, *p* = 0.0007) after returning to North Carolina following almond pollination (black) and again after blueberry pollination (gray).

Overall, lifespan was greater for bees reared in stationary colonies as compared to migratory colonies and for the trial conducted in June versus May, with the overall difference between treatments being approximately 1 day. Colony treatment (*X*^2^ = 11.39, *p* = 0.0007) and trial (*X *^2^ = 21.11, *p* < 0.0001) significantly impacted worker lifespan, but there was no interaction between the two effects (*p* > 0.5). In May, after the colonies returned from California, the lifespan (mean a number of days ± s.e.) of individuals from stationary colonies was 19.45 ± 0.32 (N = 327) and 18.01 ± 0.32 (N = 338) for bees from migratory colonies (Fig. 2). After the bees returned from conducting pollination services in Maine in June, the mean was 20.49 ± 0.35 days (N = 382) for individuals from stationary colonies and 19.89 ± 0.35 days (N = 378) from migratory colonies (Fig. 2).

### Experiment 2: Effects of experimental migratory management

#### Colony health and status

**Figure 3:**
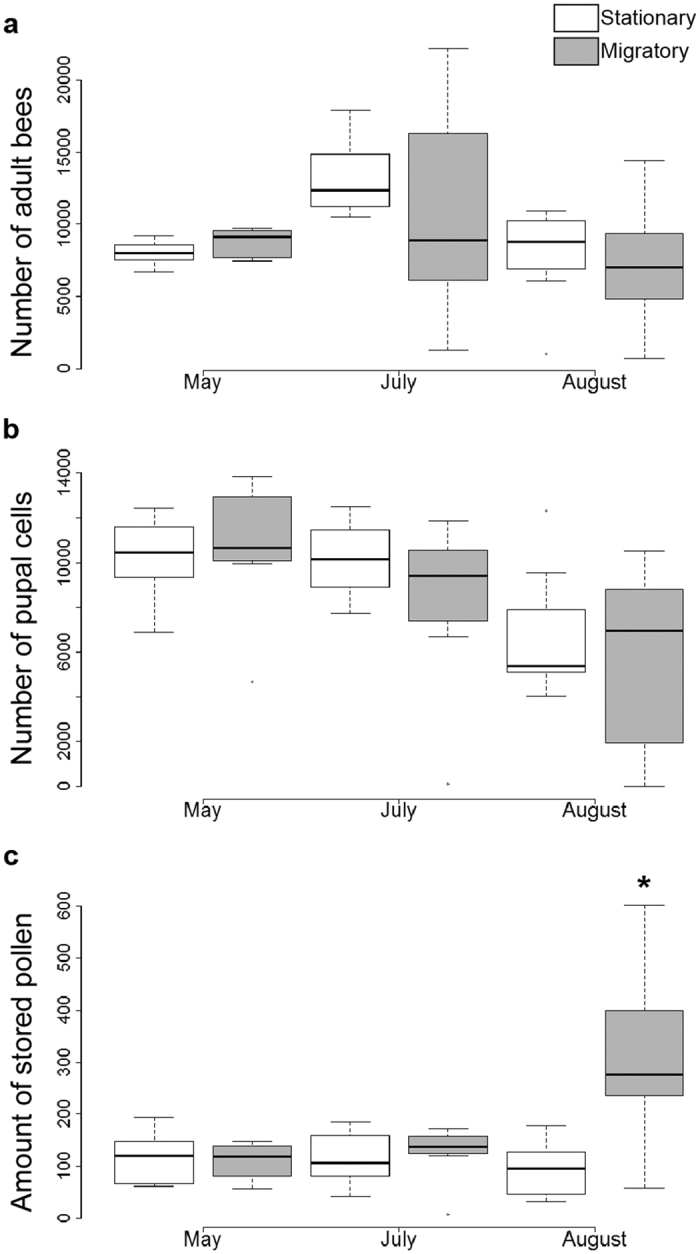
Colony health and productivity. Box plots showing the minimum, first quartile, median, third quartile, and a maximum of the following types of data from Experiment 2: (**a**) Number of adult bees per colony; (**b**) Amount of sealed pupal cells, and (**c**) Number of cells containing stored pollen. N = 7 colonies per treatment. For each measure there was a significant effect of time, but not treatment, except for pollen (**c**) where migratory colonies had more pollen significantly in August, as indicated by the star (treatment*time interaction: *F*_*2,11*_ = 0.83, *p* = 0.03).

Demographic data was normally distributed based on the goodness of fit test (Shapiro-Wilk W test; JMP Pro 10). For the number of adult bees and amount of sealed brood, there was an effect of time alone (*p* = 0.008 and *p* = 0.003, respectively). However, the amount of stored pollen was different with respect to the amount of pollen collected across the two colony treatments over the study period, with migratory colonies having more stored pollen than stationary colonies at the final time point (*F*_*2,11*_ = 0.83, *p* = 0.03, Fig. 3c). There were no statistical differences in the numbers of adult bees (F1,11 = 0.08, p = 0.6; Fig. 3a), and no interaction between colony type and time of measurement. There were no statistical differences of sealed brood either (*F*_*1,11*_ = 0.28, *p* = 0.26, Fig. 3b), and no interaction with treatment over time.

There was no difference in number of mites at the end of the experiment, though there was a non-significant trend for percentage of mite infestation (*p* = 0.17) to be higher in migratory (17.8%) versus stationary (10.3%) colonies, with the variance being slightly higher in migratory colonies (Levene test for unequal variance: *p* = 0.06, Figure S1).

#### Lifespan

**Figure 4:**
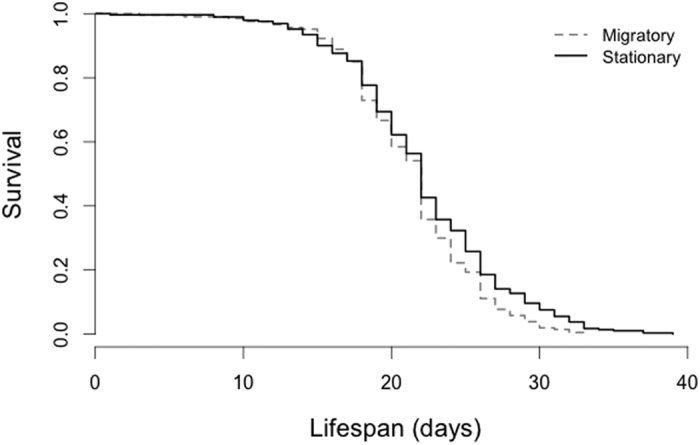
In Experiment 2, bees reared as larvae in migratory colonies (dashed line) had decreased survival compared to those reared in stationary colonies (solid line) based on ~500 individuals collected from 7 colonies per treatment (*X*^2^ = 6.48, *p* = 0.011).

The mean lifespan (number of days ± s.e.) of workers from stationary colonies (22.19 ± 0.32; N = 291) was about 1 day greater than that of workers from migratory colonies (21.34 ± 0.32; N = 207; *X*^2^ = 6.48, *p* = 0.011, Fig. 4). This finding was consistent with the results of Experiment 1.

#### Oxidative stress (lipid peroxidation)

**Figure 5:**
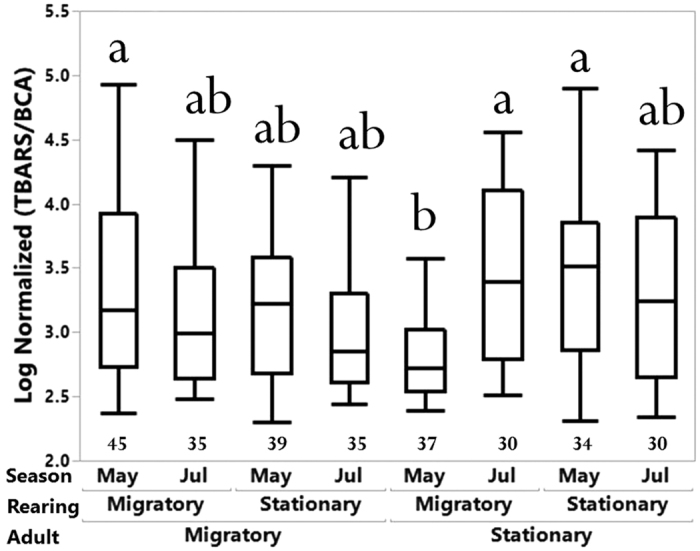
Comparison of an oxidative stress biomarker between migratory and stationary bees based on the factors of adult environment, rearing environment, and season in Experiment 2. Numbers below box-plots are the number of bees tested for each category. Bars that do not share a common letter above them differ at *p* < 0.05.

Log transformation was applied to the data in order to have a normal distribution and positive values. Although some significant group differences were observed (Fig. 5), no significant overall effects were detected for age (*F*_*1,272*_ = 1.30, *p* = 0.25), adult environment (*F*_*1,272*_ = 1.08, *p* = 0.30), rearing environment (*F*_*1,272*_ = 0.18, *p* = 0.67), season (*F*_*1,272*_ = 0.42, *p* = 0.52), or collection type (*F*_*1,272*_ = 0.27, *p* = 0.60). However, significant interactions were detected for “adult environment x season” (*F*_*1,272*_ = 8.19, *p* < 0.005), “rearing environment x season” (*F*_*1,272*_ = 6.19, *p* < 0.05), and “adult environment x rearing environment x season” (*F*_*1,272*_ = 7.68, *p* < 0.01; see Fig. 5).

### Experiment 3: Effect of intensive migratory management

**Figure 6:**
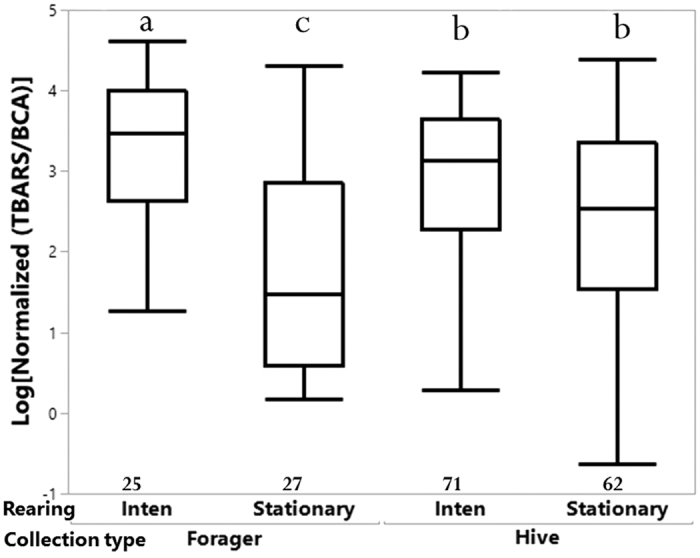
Comparison of an oxidative stress biomarker between intensive migratory (Inten) and stationary bees based on the factors of rearing environment and collection type in Experiment 3. Foragers in the intensive migratory hives as rearing environment experienced the highest level of lipid peroxidation. The number below the box-plot is the total amount of individual bees tested (N) for each category. Bars that do not share a common letter above them differ at *p* < 0.05.

Significant impacts on oxidative stress were detected for rearing environment (*F*_*1,183*_ = 35.69, *p* < 0.001), “rearing environment x collection type” (*F*_*1, 183*_ = 24.35, *p* < 0.001), and “age x collection type” (*F*_*1, 183*_ = 8.01, *p* < 0.01; see Fig. 6).

## Discussion

One hypothesis that has been put forth to explain why beekeeping operations are experiencing increased losses compared to historical rates^30^ is the stress placed upon bees from being overworked during pollination of agricultural systems. It is plausible to assume that transportation among agricultural landscapes imposes stress on colonies. Our results show that worker bees in colonies that are moved exhibit increased levels of stress, as evidenced by the fact that bees from migratory colonies (both those reared in a commercial operation and those that we controlled) had significantly reduced lifespans as compared to those from stationary colonies.

If migratory colonies experience more stress than stationary ones and if the bees had been aged in their hives rather than the controlled incubator setting, it is possible that this effect on lifespan could actually be more significant. While a difference of only 1 day may seem relatively trivial, it represents approximately 5% of the total lifespan of an adult worker and maybe up to 20% of their foraging lifespan^31^. Indeed, studies have shown that a small reduction in lifespan can exacerbate colony decline by inducing precocious foraging of surviving bees, which in turn increases their mortality rate that can escalate until the colony declines^32^. Though we did not see significant reductions in colony populations through the course of our study, this might have been the case if we had followed these colonies through winter or possibly could have been mitigated by the nutritional boost seen in the migratory colonies at the end of the season (e.g. increased pollen stores).

Our findings of stress levels, the accumulation of oxidative damage as measured by MDA levels, showed more complex effects from migration than lifespan alone. Levels of oxidative damage are often correlated to aging^18^,^19^. Early in the season, we observed that worker bees reared in a migratory colony environment and then placed in a stationary colony environment as adults had reduced levels of oxidative damage compared to those maintained in migratory colonies their entire lives. This suggests that increases in oxidative stress as a result of moving among agricultural landscapes within a relatively short term may have significant effects on colony health and productivity, either through direct effects on the stressed individuals or indirect effects on their ability to rear the subsequent generation of larvae. Given that the effects on individual bee development and physiology have only been previously documented 24-hours after transportation^10^, this is the first report of measurable long-term effects of migratory beekeeping on an important aspect of colony health.

Nonetheless, this increase in oxidative damage was not apparent later in the season. In fact, levels of MDA increased from early- to late- season for bees in stationary colonies, whereas levels were consistent for bees in migratory colonies. While bees in agricultural landscapes have the potential for increased exposure to pesticides (which can induce oxidative stress^33^), they may also have increased food resource availability even though those resources may be largely in a monoculture. In the Piedmont area of North Carolina, where this study was conducted, few food sources are available in July and August outside of agricultural landscapes. The bees in our stationary environment were, therefore, likely working harder to find scarcer food resources than those in the migratory environment (which had a ready supply of resources from mid-season crops such as soybeans and cotton later in the season, see Tables S1 and S2). This is evidenced by the increase in the amount of stored pollen seen in migratory versus stationary colonies at the final time point of assessment. The difficulty of the stationary bees to access ample forage appeared to have negated the possible benefit of remaining stationary regarding oxidative stress accumulation as seen earlier in the season. As bees are moved for pollination services, they can often have access to a ready supply of pollen or nectar sources, and even if it is of poor quality it may be better than locations where food is more limited and patchy. So far, few studies have looked into the effect of landscape on honey bee colony health^34^,^35^ and oxidative stress levels^33^. However, habitat-scale effects on physiological stress levels may be more common than anticipated^36^,^37^.

Early life experience can be a potentially significant contributor to the changes in oxidative stress of later adult life. Lipid peroxidation measures via MDA of both migratory (Experiment 2) and intensive migratory management (Experiment 3) indicated the importance of rearing conditions (Figs 5 and 6). Recent studies indicated that stress during the larval stages can influence the behavioral phenotype in the adult stage of honey bees and other insects^38^,^39^,^40^,^41^. The rearing condition is essential for adult worker behavior, lifespan, and physiology^42^. Specifically, pollen availability can significantly affect larval nutrition and physiology. Conditions experienced during the pupal stage can also impact adult function, as the core nest temperature has been shown to influence brain function and organization^43^. Changing rearing conditions during development may also generate social cues for different environmental signals and disrupt the circadian rhythm^44^. Furthermore, oxidative stress induced in the brain can affect aggression behaviors of worker honey bees^45^. Subsequent studies are needed to determine how developmental stress affects adult phenotype.

Our experimental hives were trucked only short distances (~1 hour in Experiment 2) compared to the typical migratory experience, where they can remain confined on a truck for 12–72 hours passing across the entire expanse of the U.S. Moreover, standard operating procedure typically has them placed in a holding yard that may not have adequate forage, rather than being directly placed in a blooming field as they were in our study. The fact that we detected effects in lifespan and stress after a relatively short migratory period (one move for the bee lifespan analysis and two moves for measures of oxidative stress in Experiment 2) indicates that this practice may further add to the challenges that bees are currently facing, as the effects we documented here are potentially an underestimate of the effects that may occur when colonies are moved longer distances. Another factor that may influence colony health due to movement of hives is the potential increase in the drifting of workers^46^. The marginal increase in the variance of mite levels in the migratory versus stationary colonies (Figure S1) and a higher proportion of painted bees found in colonies other than where they were introduced (M. Simone-Finstrom, personal observation) suggests higher drift of workers in the migratory operation^47^. However, low sample size constrains the statistical power to make strong inferences at the colony level regarding this point.

It is important to note that in recent colony loss surveys of the apicultural industry, the highest mortality was seen in “sideliner” operations (beekeepers with less than 500 colonies) that transport their bees for almond pollination and in “backyard” (i.e., stationary) operations^9^. A typical sideliner operation would, in fact, transport colonies more regionally, as we did in the current study, except almond pollination. Thus, some component of large-scale commercial beekeeping may, in fact, be mitigating the impacts of migratory stress on their bees compared to the small-scale migration of “sideliner” and stationary operations.

Now that we have determined that migratory beekeeping influences honey bee longevity and stress and that it interacts with environmental factors, future work is needed to isolate the specific stressors related to migratory management. For example, swapping collected pollen between migratory and stationary bees would help determine if nutrition or exposure to pesticides through forage explains at least some of these observed effects. Additionally, longer-term (i.e., overwintering) effects need to be investigated, and those even beyond a single season.

To mitigate the influence of migratory management on bee health, managing the local environment where colonies are kept is an increasingly important aspect to consider, including moving typically stationary colonies when resources are highly limited. This recommendation is of particular importance because supplemental feedings may not be sufficient to maintain healthy colonies^48^. In addition to moving hives, the amelioration of local habitat through the addition of hedgerows in agroecosystems and planting large-scale plots of bee-friendly plants may improve the nutritional landscape and consequently the health of honey bees. Importantly, the time of bloom of these plants needs to be considered to supply food better throughout the season. Additionally, commercial beekeeping operations, as many already do, should consider selectively transporting subsets of colonies, so that each colony is transported fewer times to limit the effect on any particular colony. Follow-up research is needed to address specifically how the frequency of trips may affect colony health and oxidative stress resistance.

The study presented here provides the first comprehensive evidence that migratory management impacts bee health and oxidative stress. Based on our results, it appears that the conditions under which individuals are reared as larvae and pupae are particularly important in regards to future resistance to oxidative stress and lifespan. It seems evident, however, that resource availability may mask these effects later in life, placing a much higher emphasis on natural forage and landscape suitability for proper developmental nutrition. Together, these aspects highlight the complexity of interactions among oxidative stress resistance, longevity, and resource allocation at both the group and individual levels.

## Additional Information

**How to cite this article**: Simone-Finstrom, M. *et al*. Migratory management and environmental conditions affect lifespan and oxidative stress in honey bees. *Sci. Rep.* **6**, 32023; doi: 10.1038/srep32023 (2016).

## Supplementary Material

Supplementary Information
