# Supplementary material for: Migratory management and environmental conditions affect lifespan and oxidative stress in honey bees
Source: Sci Rep. 2016 Aug 24;6:32023. doi: 10.1038/srep32023 (PMC4995521; doi:10.1038/srep32023)
Supplement: Supplementary Information [file srep32023-s1.pdf]

Migratory management and environmental conditions affect lifespan and oxidative stress in honey bees

Michael Simone-Finstrom[1#*], Hongmei Li-Byarlay[1, 2, 3#], Ming H. Huang[1], Micheline K. Strand[2],

Olav Rueppell[3], David R. Tarpy[1]

Supplementary Methods and Information

Experiment 2: Apiary locations

Landscape descriptions were determined by examining a 1500 m radius of each of the four apiary locations (Table S1), using the NLCD 2011 land cover dataset (Homer et al. 2015) in ArcMap 10.0 following Youngsteadt et al. 2015. A 1500 m radius represents a typical average foraging distance for *A. mellifera*. The stationary colonies were maintained in the Raleigh location, whereas the migratory colonies were moved among the 4 locations (Table S2).

Table S1. Description of the surrounding areas for the stationary and migratory apiaries in Experiment 2.

	**% Coverage at 1500m around each apiary location**			
**Landscape type**	Raleigh	Clayton	Goldsboro	Rocky Mount
Water and wetlands	5.07	2.45	20.21	21.38
Developed (open/barren land or infrastructure)	17.50	33.68	13.32	6.68
Shrubs/grassland	3.83	9.01	7.13	3.98
Forest	49.68	22.96	4.38	2.93
Crops (pasture, hay, cultivated crops)	23.92	31.90	54.95	65.03

Table S2. Date and location of migratory movements described for Experiment 2.

**Migratory movements**	**Location**	**Crops of note**
4-May-12	Central Crops Research Station, Clayton, NC	Corn planted, berries
25-May-12	Cherry Research Farm, Goldsboro, NC	Corn, various small crops
15-Jun-12	Central Crops Research Station, Clayton, NC	Watermelon, curcubits
6-Jul-12	Upper Coastal Plain Research Station, Rocky Mount, NC	Soy beans, tobacco, cotton
27-Jul-12	Central Crops Research Station, Clayton, NC	Soy beans, cotton

Experiment 2: Varroa

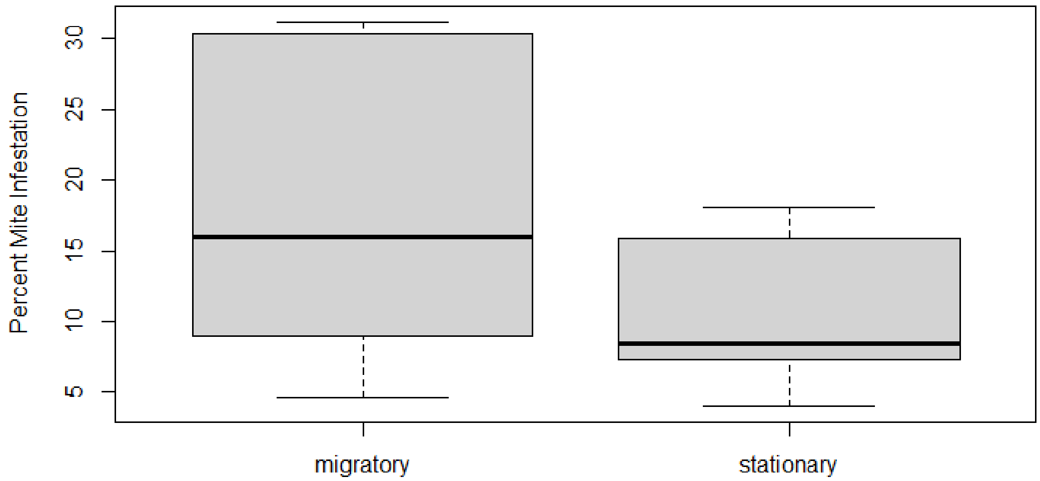

Figure S1. Percent infestation of Varroa in colonies (number of mites per 100 adult beees) at the end of Experiment 2. N = 6 for each colony treatment.

Experiment 3: Apiary locations

Table S3. Distance between and coordinates for locations described for the intensive migratory movements described in Experiment 3.

Location	Distance to next site (km)	Approx. Coordinates
Back 40 (Raleigh, NC)	338	35.724619, -78.700059
Shelby, NC	329	35.293423, -81.533955
Varsity Drive (Raleigh, NC)	344	35.779161, -78.683669
Williamsburg, VA	344	37.255171, -76.704604
Fish Barn (Raleigh, NC)	376	35.728024, -78.672124
Black Mountain, NC	373	35.617434, -82.316104
Hops (Raleigh, NC)		35.736935, -78.685817

References

Homer, C.G., Dewitz, J.A., Yang, L., Jin, S., Danielson, P., Xian, G., Coulston, J., Herold, N.D., Wickham, J.D., and Megown, K. (2015) Completion of the 2011 National Land Cover Database for the conterminous United States-Representing a decade of land cover change information. *Photogrammetric Engineering and Remote Sensing*, 81(5): 345-354

Youngsteadt E, Appler RH, López-Uribe MM, Tarpy DR, Frank SD (2015) Urbanization Increases Pathogen Pressure on Feral and Managed Honey Bees. *PLoS ONE* 10(11): e0142031. doi: 10.1371/journal.pone.0142031
